# Case report: acute isolated cilioretinal artery occlusion secondary to percutaneous coronary intervention

**DOI:** 10.1186/s13019-023-02379-y

**Published:** 2023-10-17

**Authors:** Bangtao Yao, Zhaoyang Dong, Yuanfeng Xue, Haoyu Meng, Fei Wang

**Affiliations:** 1 Department of Ophthalmology, Nanjing Lishui People’s Hospital, Zhongda Hospital Lishui branch, Southeast University, Nanjing, Jiangsu Province China; 2 Department of Ophthalmology, Nanjing Jiangning Hospital of Chinese Medicine, Nanjing, Jiangsu Province China; 3 Department of General Practice, Nanjing Lishui District Baima Health Hospital, Nanjing, Jiangsu Province China; 4 Department of Cardiology, Jiangsu Province Hospital, The First Affiliated Hospital of Nanjing Medical University, Nanjing, Jiangsu Province China; 5 Department of Ophthalmology, Jiangsu Province Hospital, The First Affiliated Hospital of Nanjing Medical University, Nanjing, Jiangsu Province China

**Keywords:** Cilioretinal artery occlusion, Coronary artery disease, Percutaneous coronary intervention, BCVA, *Salvia miltiorrhiza*

## Abstract

**Introduction:**

This case report aims to describe in detail the acute isolated cilioretinal artery occlusion (CLRAO) secondary to complicated therapeutic percutaneous coronary intervention (PCI).

**Case description:**

A 68-year-old Chinese man with coronary artery disease (CAD) complained of sudden, sharp chest pain. Coronary angiography revealed severe stenoses of the coronary arteries. The patient was then treated with PCI. One hour after the procedure, the patient presented with a sudden reduction in vision in the right eye. The patient was diagnosed with acute isolated CLRAO and treated with *Salvia miltiorrhiza* injections.

**Conclusions:**

This is the report to provide a detailed description of acute isolated CLRAO secondary to therapeutic PCI treated with *Salvia miltiorrhiza*. The visual prognosis of the untreated patients is poor. Suitable management and prevention are essential for interventional cardiologists to prevent these complications.

**Supplementary Information:**

The online version contains supplementary material available at 10.1186/s13019-023-02379-y.

## Introduction

Coronary artery disease (CAD) is a common heart disease caused by myocardial ischemia, hypoxia and even necrosis due to the reduction of coronary artery blood flow, which leads to a high rate of morbidity and mortality worldwide [1, 2]. Although percutaneous coronary intervention (PCI) is currently indicated as an effective treatment for patients with severe coronary artery stenosis, several complications have been reported [1, 2]. To the best of our knowledge, retinal artery occlusion (RAO) secondary to PCI has been rarely reported [3].

Cilioretinal artery occlusion (CLRAO) is an acute, rare, and severe form of RAO that features sudden visual reduction and retinal ischemia in the region of the occlusive cilioretinal artery, with central retinal artery sparing [4, 5]. CLRAO is clinically divided into three subtypes: (1) isolated CLRAO, (2) CLRAO with central retinal vein occlusion, and (3) CLRAO with ischemic optic neuropathy [6, 7]. Management of acute CLRAO remains challenging.

*Salvia miltiorrhiza* (*Danshen* in Chinese), a traditional herbal medicine containing many individual compounds, has been widely used in the treatment of cardiovascular diseases in Asia [8]. Animal experiments have shown that *Salvia miltiorrhiza* can pass through the blood-ocular barrier and improve retinal microcirculation [9]. However, its application to RAO has not been reported. This case report highlights the treatment of a rare case of acute isolated CLRAO secondary to PCI, treated with *Salvia miltiorrhiza*.

## Case description

**Fig. 1:**
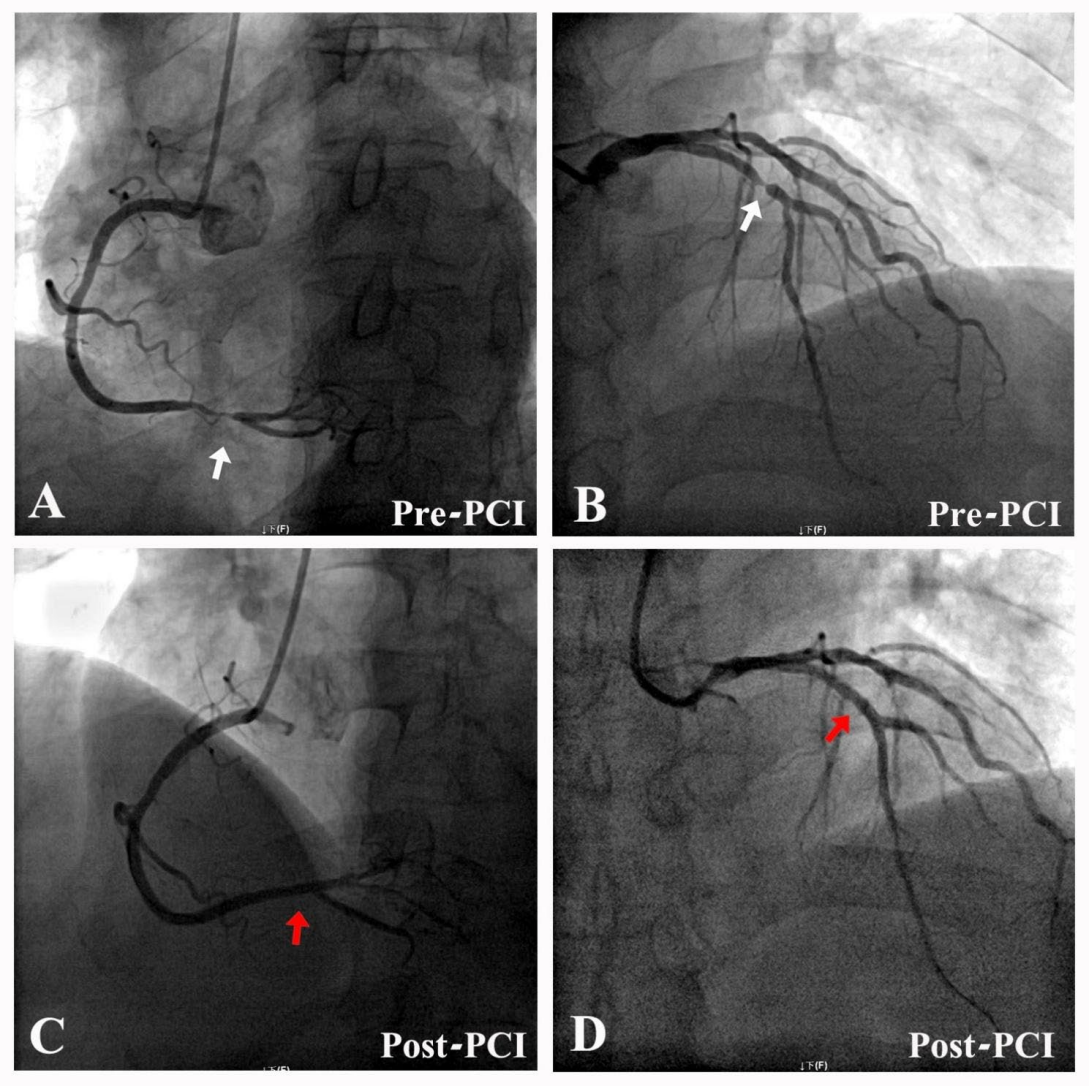
Coronary angiography showed severe stenosis of the distal right coronary artery (**A**) and the proximal left anterior descending coronary artery (**B**). After PCI, the stenosis were released by two stents implantation, respectively (**C**, **D**)

A 68-year-old Chinese man complained of sudden, sharp chest pain that had commenced 2 days prior to presentation. He was finally diagnosed as unstable angina, accompanied by hypertension, diabetes mellitus and hyperlipidemia. Coronary computer tomography angiography and two-dimensional echocardiography showed aortic atherosclerosis and no evidence of cardio-embolic phenomenon. Coronary angiography showed severe stenosis of the distal right coronary artery (Fig. 1A) and the proximal left anterior descending coronary artery (Fig. 1B). After receiving a detailed written informed consent, the patient was treated with PCI. Anti-platelet therapy was loaded with aspirin (300 mg) and clopidogrel (300 mg) before PCI. During PCI, anticoagulation was performed using intravascular heparin with 100 U/kg, and activated coagulation time was monitored and controlled between 250 and 350 s (heparinization). Low-molecular-weight heparin was used for 3 days after PCI. After PCI, the stenosis of the affected arteries were released by two stents implantation, respectively (Fig. 1C, D). Chest pain was significantly alleviated. One hour after the procedure, the patient presented with a painless sudden reduction in vision in the right eye; however, he sought ophthalmic consultation until the next morning.

**Fig. 2:**
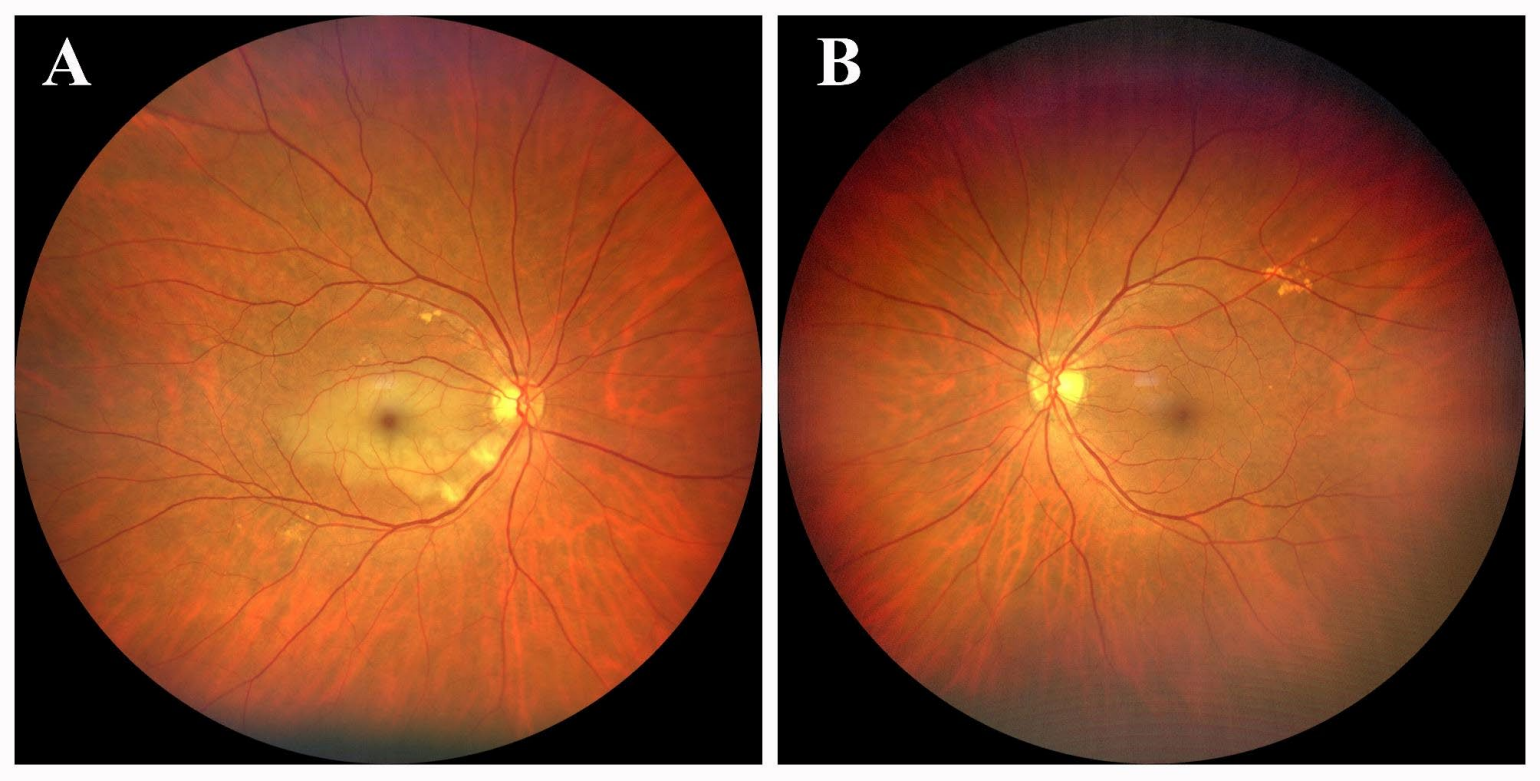
Funduscopic examination revealed the tongue-shaped retinal infarct in the region of the occlusive cilioretinal artery, retinal oedema, and cotton-wool spots in the right eye, the optic disc, retinal branch arteries, and retinal branch veins appeared normal (**A**). The fundus of the left eye was unremarkable (**B**)

**Fig. 3:**
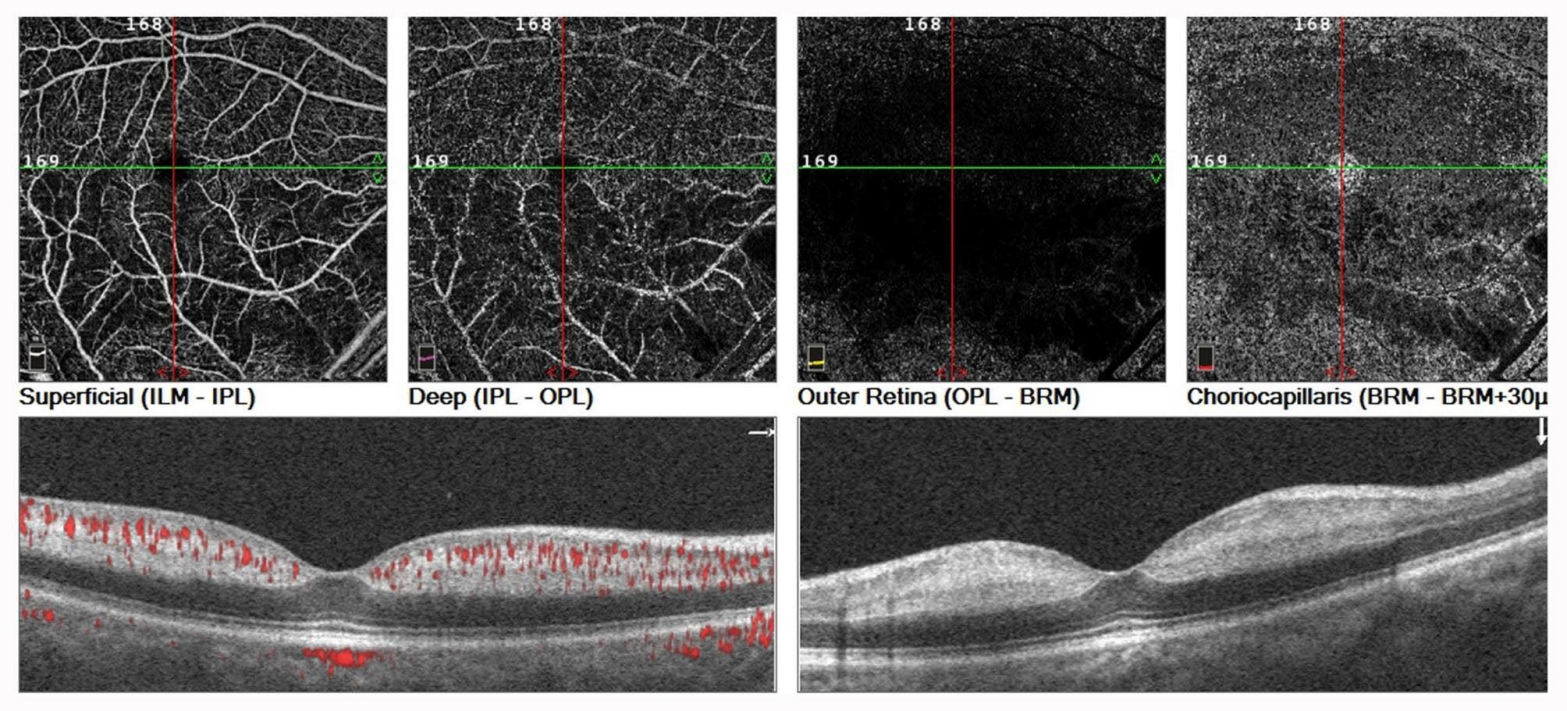
OCTA of the right eye showed the decreased flow sign in the superficial, deep retinal layers that extends from the optic disc to the whole macula, the outer retinal layer appeared normal, the artifact of the decreased density of the choroidal vasculature was observed in the choroid capillary layer. OCT B-san demostrated the hyper-reflective thickening of the entire inner retina involving the fovea

**Fig. 4:**
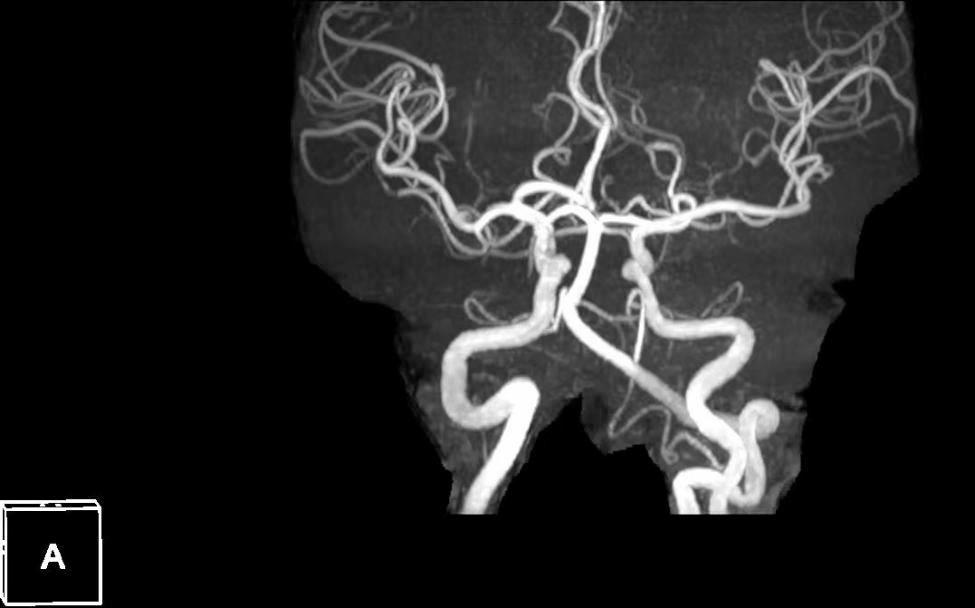
Postoperative intracranial magnetic resonance angiography showed no new onset infarct focus

The best corrected visual acuity (BCVA) was calculated by counting the fingers at 50 cm oculus dexter and 20/20 oculus sinister. The corneas were clear. Pupils were equal, round, and reactive to light with no afferent pupillary defect. Funduscopic examination revealed a tongue-shaped retinal infarct in the cilioretinal artery, retinal edema, and cotton-wool spots in the right eye (Fig. 2A). The fundus of his left eye was unremarkable (Fig. 2B). Optical coherence tomography angiography (OCTA) of the right eye in the fovea showed a decreased flow sign in the superficial and deep retinal layers, extending from the optic disc to the whole macula. OCT B-scan demonstrated hyperreflective thickening of the inner retina, involving the fovea (Fig. 3). The peripheral visual field appeared normal. Two-dimensional echocardiography performed after the operation revealed no cardio-embolic evidence. Postoperative carotid ultrasound found atherosclerotic plaques in the right carotid artery, and intracranial magnetic resonance angiography showed no new onset infarct focus (Fig. 4). The peri-operative electrocardiogram monitoring captured no evidence of cardiac arrhythmia, and perioperative echocardiography had eliminated valvular heart disease. His blood pressure was 134/86 mmHg. Laboratory results including erythrocyte sedimentation rate, blood glucose, and serum lipids were unremarkable. He refused to undergo fundus fluorescein angiography. Based on the above findings, he was definitively diagnosed with acute, isolated CLRAO.

The patient requested for conservative treatments and was treated with 10 mL *S. miltiorrhiza* injection once daily for 2 weeks. Oral aspirin (100 mg/day) and clopidogrel (75 mg) were suggested for maintaining; and blood pressure, blood glucose, and serum lipid levels were monitored.

During the two-week follow-up, his BCVA improved to 20/400; however, the fundus appeared similar to that at the time of onset.

## Discussion

Although PCI is currently proven effective for the management of patients with coronary artery stenosis secondary to CAD, which is associated with diabetes mellitus, hyperlipidemia, and hypertension, several complications, including coronary perforation, abrupt vessel closure, arrhythmia, and embolization, have been reported [1, 2]. However, the mechanism underlying RAO secondary to PCI remains unknown.

In Western medicine, RAO is treated to be an ischemic stroke with an incidence of 0.85/100,000 per year [10]. CLRAO is less common (5%) and is characterized by retinal ischemia in the corresponding supply area with central retinal artery sparing [11]. CLRAO is closely associated with carotid artery stenting [5]. However, CLRAO secondary to PCI is very rare, and only two cases of CLRAO following diagnostic coronary angiography have been reported [11, 12]. In this case report, we speculate that the plaque released from the brachiocephalic trunk during the perioperative period of PCI went through the internal carotid artery and blocked the ciliary retinal artery. Adequate fluoroscopy tracing was required when the guide wire or catheter passed through the brachiocephalic trunk during radial PCI to prevent any device from entering the internal carotid artery, especially for patients with carotid plaques indicated by preoperative carotid ultrasound. If any difficulty occurs while passing through the brachiocephalic trunk, the hydrophilic coating wire or percutaneous transluminal coronary angioplasty wire may work by advancing it gently in coordination with the breathing of the patients, failing which the femoral artery route should be considered for performing PCI.

Untreated cases of CLRAO will result in irreversible impairment in vision [4, 5, 7]. The purpose of intervention is to restore the retinal blood supply, promote the recovery of ganglion cells, and improve visual outcomes [13]. Therefore, early diagnosis and intervention are crucial. However, the time window for an effective treatment is narrow. A scientific statement from the American Heart Association documented that intraarterial/intravenous recombinant tissue-type plasminogen activator may be effective for patients presenting within 4.5 h from symptom onset. Due to a lack of evidence for efficacy and potential harm, the conservative treatments, such as ocular massage, topical intraocular pressure lowering drugs and systemic β-blockade, have not been endorsed in current professional guidelines [14]. Besides, hyperbaric oxygen therapy is supposedly useful and beneficial for retinal tissue, and improves visual outcomes in acute RAO; however, further study is required to better understand the effects of the treatment [14].

*Salvia miltiorrhiza*, a traditional Chinese herbal medicine, reportedly has the potential to dilate vessels, improve microcirculation, and treat ischemic cardiovascular diseases, such as coronary heart disease and ischemic stroke [8]. Animal experiments have shown that *Salvia miltiorrhiza* can pass through the blood-ocular barrier and improve retinal microcirculation [9]. In our patient, his vision improved after treatment with *Salvia miltiorrhiza*; however, the retinal infarct and edema were not resolved. We concluded that the entire macular capillary network was seriously damaged, and that the treatment was outside the time window.

Differential diagnoses, such as central/branch RAO, ocular ischemic syndrome, and retinal vein occlusion, should be carefully addressed. The clinical history and features distinguish CLRAO from the aforementioned diseases. In the present patient, the fundus showed the normal appearance of the central/branch retinal arteries and veins, and no evidence of cherry red spot and retinal hemorrhage, which rule out the diagnosis of central/branch RAO and retinal vein occlusion. Besides, ocular ischemic syndrome is a severe painful ocular disorder related to ophthalmic artery hypoperfusion, the condition affected both the anterior and the posterior segments, which is distinguished from CLRAO.

However, lacking of fundus autofluorescence and fluorescein angiography is probably the main limitation of this study.

In conclusion, we present a rare case of CLRAO secondary to complicated therapeutic PCI treated with *Salvia miltiorrhiza*. This case report highlights the importance of suitable management and preventive strategies for interventional cardiologists to prevent complications. Untreated patients may have irreversible visual impairment. More data are needed to better understand the effects of *Salvia miltiorrhiza* on ischemic retinopathy.

### Electronic supplementary material

Below is the link to the electronic supplementary material.

Supplementary Material 1

Supplementary Material 2

## Data Availability

The datasets presented in this study are included in the article, further inquiries can be directed to the corresponding author/s.

## Abbreviations

BCVA: best-corrected visual acuity
CLRAO: cilioretinal artery occlusion
OCTA: Optical coherence tomography angiography
PCI: percutaneous coronary intervention
